# FAM21 directs SNX27–retromer cargoes to the plasma membrane by preventing transport to the Golgi apparatus

**DOI:** 10.1038/ncomms10939

**Published:** 2016-03-09

**Authors:** Seongju Lee, Jaerak Chang, Craig Blackstone

**Affiliations:** 1 Cell Biology Section, Neurogenetics Branch, National Institute of Neurological Disorders and Stroke, National Institutes of Health, Bethesda, Maryland 20892, USA; 2 Department of Biomedical Sciences, Ajou University School of Medicine, Suwon 443-380, Korea; 3 Department of Brain Science, Ajou University School of Medicine, Suwon 443-380, Korea; 4 Neuroscience Graduate Program, Ajou University School of Medicine, Suwon 443-380, Korea

## Abstract

The endosomal network maintains cellular homeostasis by sorting, recycling and degrading endocytosed cargoes. Retromer organizes the endosomal sorting pathway in conjunction with various sorting nexin (SNX) proteins. The SNX27–retromer complex has recently been identified as a major endosomal hub that regulates endosome-to-plasma membrane recycling by preventing lysosomal entry of cargoes. Here, we show that SNX27 directly interacts with FAM21, which also binds retromer, within the Wiskott–Aldrich syndrome protein and SCAR homologue (WASH) complex. This interaction is required for the precise localization of SNX27 at an endosomal subdomain as well as for recycling of SNX27-retromer cargoes. Furthermore, FAM21 prevents cargo transport to the Golgi apparatus by controlling levels of phosphatidylinositol 4-phosphate, which facilitates cargo dissociation at the Golgi. Together, our results demonstrate that the SNX27–retromer–WASH complex directs cargoes to the plasma membrane by blocking their transport to lysosomes and the Golgi.

## Abstract (web summary)

Endosomes maintain cellular homeostasis by sorting, recycling and degrading endocytosed cargoes. Here the authors show that the SNX27-retromer-WASH complex acts as a hub to direct cargoes to the plasma membrane by blocking their transport to lysosomes and Golgi apparatus.

## Graphical abstract

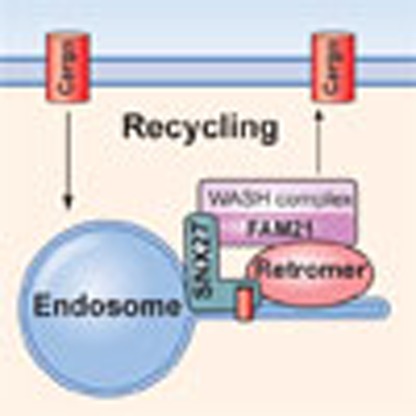

The endosomal network controls numerous cellular functions such as signalling, nutrient uptake and development through balanced trafficking of diverse plasma membrane proteins^1^,^2^. After receiving membrane protein cargoes, endosomes sort them to lysosomes for degradation or else to the *trans*-Golgi network (TGN) or plasma membrane for recycling^3^,^4^. This endosomal sorting pathway is elaborately regulated by retromer and its interacting proteins^5^,^6^. Retromer, a multi-subunit complex that associates with the cytosolic face of endosomes, was originally identified for its ability to regulate retrograde transport from endosomes to the TGN^7^,^8^,^9^,^10^. Recent studies have expanded its regulatory role in not only endosome-to-TGN transport but also in endosome-to-plasma membrane transport^6^,^11^.

Retromer comprises a core trimer of vacuolar protein sorting-associated protein 35 (VPS35), VPS26 and VPS29 as well as sorting nexin (SNX) proteins that bind phosphoinositides^12^. Although the composition of the VPS trimer is conserved, it forms complexes with various SNX proteins. Interactions of the VPS trimer with SNX1/2 and SNX5/6 regulate retrograde transport of cation-independent mannose-6-phosphate receptors from endosomes to the TGN^13^. For retrograde transport of Wntless to the TGN, retromer associates with SNX3 (ref. 14). More recently, the retromer complex associated with SNX27, a PDZ-domain-containing SNX protein, has been shown to regulate endosome-to-plasma membrane recycling of membrane proteins including the β2-adrenergic receptor (β2AR) and the glucose transporter GLUT1 (refs 6, 11). Thus, SNX proteins appear to facilitate the cargo specificity of retromer^15^,^16^. However, mechanisms for how retromer specifies destinations of individual cargoes remain unclear.

The Wiskott–Aldrich syndrome protein and SCAR homologue (WASH) complex is a large, multiprotein complex consisting of family with sequence similarity 21 (FAM21), strumpellin, SWIP, CCDC53 and WASH1; it activates Arp2/3-mediated actin polymerization on endosomes^17^,^18^,^19^. WASH complex depletion causes endosomal sorting defects in pathways such as recycling of transferrin (Tf) receptor and integrins to the plasma membrane as well as retrograde transport of cation-independent mannose-6-phosphate receptor to the Golgi apparatus^20^,^21^,^22^. Given that both SNX27 and retromer bind the WASH complex, the WASH complex has been suggested to function in recycling of SNX27–retromer cargoes, although a specific role has not been identified^6^,^23^.

Here, we have identified an interaction of SNX27 with FAM21, the largest WASH component, which is required for proper recycling of SNX27–retromer cargoes. This interaction restricts SNX27 to an endosomal subdomain, aiding export of cargoes to their destinations. Moreover, FAM21 prevents cargoes from being transported to the Golgi apparatus by controlling Golgi levels of phosphatidylinositol 4-phosphate [PI(4)P], which facilitates a release of retromer cargoes around the Golgi.

## Results

### FAM21 interacts directly with SNX27

**Figure 1:**
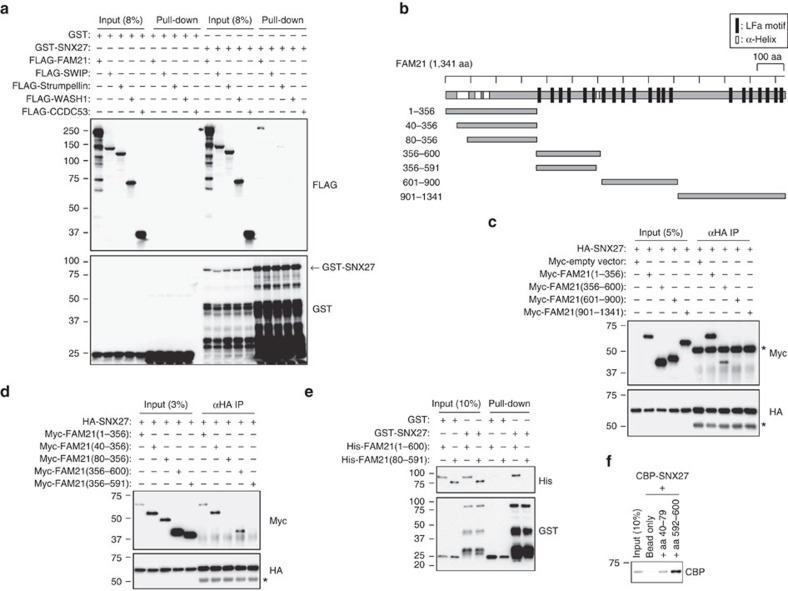
SNX27 interacts directly with FAM21 among the WASH complex components. (**a**) HEK293T cells were transfected with 3 × FLAG-tagged-WASH complex proteins. Lysates were incubated with GST or GST-SNX27 proteins and precipitated with Glutathione Sepharose 4B beads. Precipitates were then immunoblotted as shown. (**b**) Schematic representation of FAM21 constructs used. LFa motifs and α-helices are indicated. (**c**,**d**) HA-SNX27 was co-transfected with the indicated Myc-tagged constructs in HEK293T cells, and lysates were immunoprecipitated with anti-haemagglutinin (HA) antibodies and immunoblotted. Asterisks (*) denote IgG heavy chains. (**e**) GST or GST-SNX27 fusion proteins were incubated with indicated His-tagged FAM21 proteins and precipitated using Glutathione Sepharose 4B beads. Precipitates were immunoblotted as indicated. (**f**) Biotinylated peptides corresponding to FAM21 residues 40–79 and 592–600 were incubated with CBP-SNX27 and precipitated using streptavidin-conjugated beads. Precipitates were immunoblotted for CBP.

To identify the WASH complex component that binds SNX27 directly, we conducted *in vitro* pull-down assays. Among the five core proteins of the WASH complex, only FAM21 interacted with SNX27 fusion proteins purified from bacteria (Fig. 1a). FAM21 contains an N-terminal globular head domain and a C-terminal unstructured tail harbouring multiple L-F-[D/E]_3-10_-L-F (LFa) motifs (Fig. 1b); it regulates the WASH complex by interacting with various proteins through its different motifs. FAM21 links the WASH complex to endosomes via retromer binding and inhibits actin-capping activity by binding the CAPZ heterodimer via its C-terminus; the FAM21 N-terminus is required for binding within the WASH complex to maintain its stability^17^,^22^,^23^,^24^. It is thus important to determine the region of FAM21 responsible for binding SNX27. We found that SNX27 binds two distinct regions within the N-terminal domain of FAM21 (Fig. 1c). Using additional approaches including co-immunoprecipitation (Fig. 1d), glutathione *S*-transferase (GST)-pulldowns (Fig. 1e) and peptide-binding assays (Fig. 1f), we determined that residues 40–79 and 592–600 of FAM21 are sufficient for binding SNX27 directly.

### Binding of FAM21 with SNX27 mediates cargo recycling

**Figure 2:**
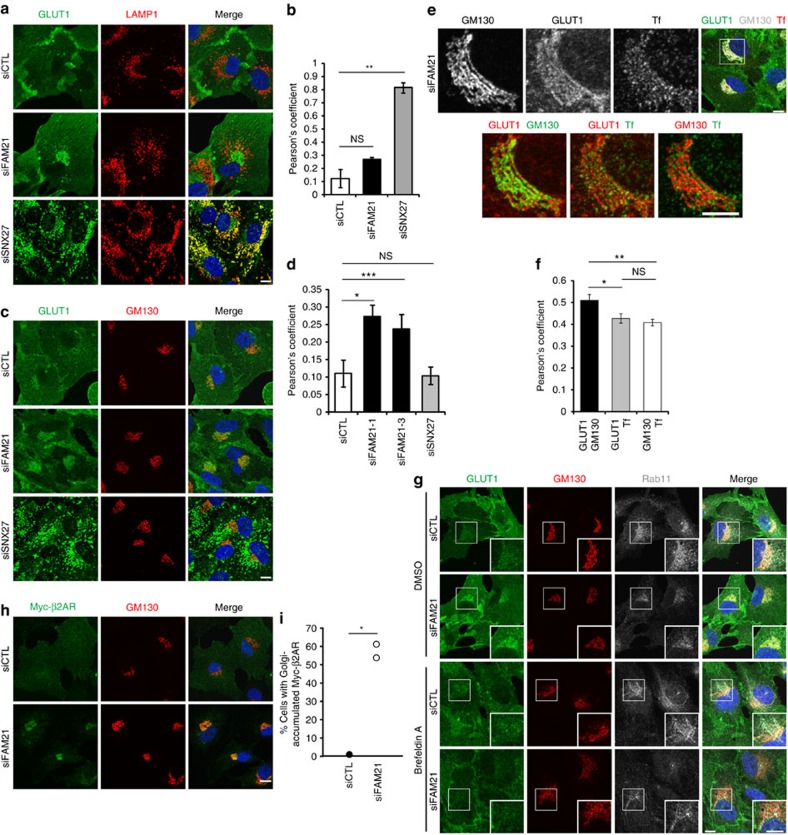
Depletion of FAM21 causes mis-sorting of SNX27–retromer cargoes to the Golgi apparatus. (**a**–**d**) hTERT-RPE1 cells transfected with the indicated specific or control (siCTL) siRNAs were immunostained for GLUT1 (green) along with LAMP1 (**a**, red) or GM130 (**c**, red). Co-localization of GLUT1 with LAMP1 (**b**) or GM130 (**d**) was analysed by calculation of Pearson's coefficient. Graphs express means±s.d. (*n*=3; >30 cells per group). (**e**) hTERT-RPE1 cells transfected with an siRNA targeting FAM21 were incubated with 10 μg ml^−1^ of Alexa 568-conjugated Tf for 20 min at 37 °C before fixation. Cells were then immunostained for GM130 and GLUT1. For comparison, representative magnified views of the Golgi were chosen and displayed. (**f**) Co-localization at the Golgi area among GLUT1, GM130 and Tf were analysed by calculation of Pearson's coefficient. Graphs express means±s.d. (30 cells per group). (**g**) hTERT-RPE1 cells transfected with the indicated siRNAs were incubated with 0.05% of dimethylsulphoxide or 5 μg ml^−1^ of Brefeldin A for 5 min at 37 °C before fixation and immunostained for GLUT1 (green), GM130 (red) and Rab11 (grey). Insets are magnified views of the Golgi. (**h**) hTERT-RPE1 cells stably expressing Myc-β2AR were transfected with the indicated siRNAs and immunostained for Myc epitope (green) and GM130 (red). (**i**) Cells with Myc signals accumulating at the Golgi from **h** were counted (*n*=2; 200 cells per group). Merged images with 4',6-diamidino-2-phenylindole staining (blue) are to the right. Scale bars, 10 μm. *P<0.05, **P<0.01, ***P<0.001; NS, not significant.

The WASH complex has been suggested to be involved in endosome-to-plasma membrane transport mediated by the SNX27-retromer complex^6^,^11^. To investigate whether FAM21 functions in recycling of SNX27–retromer cargoes, we examined the localization of GLUT1, a known cargo of the SNX27–retromer complex, after small interfering RNA (siRNA) transfection. As previously reported^6^, depletion of SNX27 resulted in GLUT1 accumulation at LAMP1-positive lysosomes (Fig. 2a,b). Intriguingly, GLUT1 accumulated in the perinuclear region, not at lysosomes, in FAM21-depleted cells (Fig. 2a,b). Immunofluorescence staining of GM130, a *cis*-Golgi matrix protein, revealed that depletion of FAM21 caused a selective accumulation of GLUT1 in the Golgi region (Fig. 2c,d). This finding was observed using two different siRNA sequences targeting FAM21 (Fig. 2d and Supplementary Fig. 1), and thus it is likely not an off-target effect.

The mis-sorting of GLUT1 to the endocytic recycling compartment, which is adjacent to the Golgi apparatus, was recently reported^25^. To determine the precise location of mis-sorted GLUT1, we carefully analysed the immunofluorescent signals of GLUT1, GM130 and Tf near the Golgi in FAM21-depleted cells. The staining pattern of GLUT1 was continuous, like GM130, rather than more punctate, like Tf (Fig. 2e). Also, the co-localization coefficient of GLUT1 with GM130 was significantly higher than it was with Tf (Fig. 2f). The mis-sorted GLUT1 disappeared after treatment of brefeldin A (BFA), which dissociates Golgi membrane-associated proteins^26^,^27^,^28^, indicating that GLUT1 was associated with the Golgi membranes (Fig. 2g). The GLUT1 released from the Golgi membranes by BFA did not localize to Rab11-positive recycling endosomal tubules, which were also induced by BFA treatment^29^ (Fig. 2g). Together, these results suggest that GLUT1 is mis-sorted to the Golgi apparatus in FAM21-depleted cells.

To examine recycling of another SNX27-retromer cargo, we generated hTERT-RPE1 cells stably expressing β2AR. The β2AR also accumulated at the Golgi apparatus in FAM21-depleted cells (Fig. 2h,i). Mis-sorting defects induced by FAM21 depletion seemed specific to SNX27–retromer cargoes because the localization of Na,K-ATPase, a transmembrane protein not affected by SNX27 depletion, was unchanged in FAM21-depleted cells (Supplementary Fig. 2). These results indicate that FAM21 is involved in recycling of SNX27–retromer cargoes. However, the fact that GLUT1 is mis-sorted into a different intracellular compartment upon FAM21 depletion, as compared with SNX27 or retromer depletion, implies a distinct role for FAM21 with retromer in the recycling pathway.

**Figure 3:**
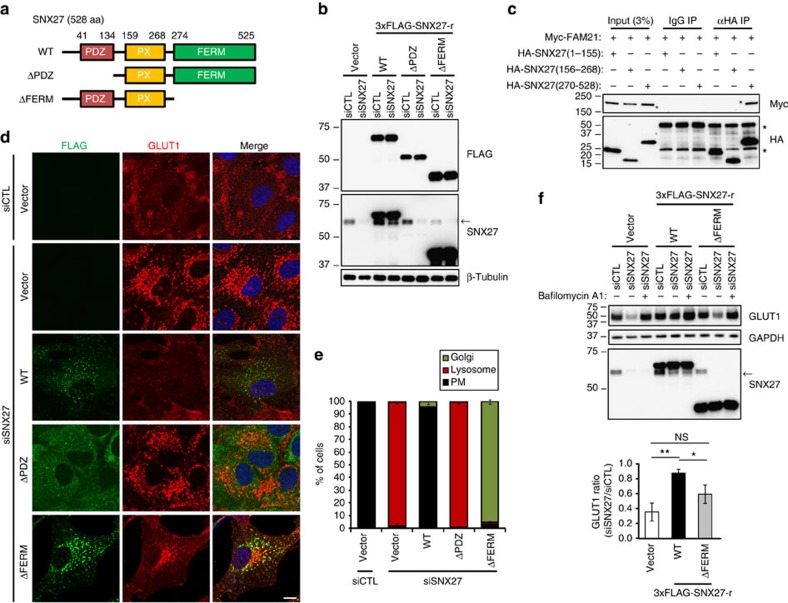
The interaction of SNX27 with FAM21 is required for proper recycling of its cargo to the plasma membrane (PM). (**a**) Schematic representation of SNX27 constructs used. (**b**) hTERT-RPE1 cells stably expressing empty vector or 3 × FLAG-tagged, siRNA-resistant SNX27 constructs were transfected with indicated SNX27 or control (siCTL) siRNAs, and analysed by immunoblotting. An arrow denotes endogenous SNX27. The SNX27 ΔPDZ proteins were not detected by anti-SNX27 antibodies because of a deficiency of epitope. (**c**) Myc-FAM21 was co-transfected with the indicated HA-SNX27 constructs in HEK293T cells, and lysates were immunoprecipitated with control mouse IgG or anti-haemagglutinin (HA) antibodies. Then, precipitates were immunoblotted as indicated. Asterisks (*) denote IgG heavy or light chains. (**d**) hTERT-RPE1 cells stably expressing empty vector or 3 × FLAG-tagged, siRNA-resistant SNX27 constructs were transfected with the indicated siRNAs and immunostained for FLAG epitope (green) and GLUT1 (red). Merged images with 4',6-diamidino-2-phenylindole staining (blue) are to the right. Scale bar, 10 μm. (**e**) Cells were counted based on co-localization of GLUT1 with GM130 (Golgi) or LAMP1 (lysosome) as shown in Supplementary Fig. 3. Cells without accumulated GLUT1 signals were considered as plasma membrane (PM). Graphs express means±s.d. (*n*=3; 200 cells per group). (**f**) hTERT-RPE1 cells stably expressing empty vector or 3 × FLAG-tagged, siRNA-resistant SNX27 constructs were transfected with the indicated siRNAs. Twenty-four hours later, cells were cultured in the absence or presence of 50 nM bafilomycin A1 for 24 h, and then analysed by immunoblotting. The intensities of each GLUT1 were normalized with respective GAPDH signals. Levels of GLUT1 in siSNX27-transfected cells relative to siCTL-transfected cells were measured from five independent experiments. Graphs express means±s.d. *P<0.05, **P<0.01; NS, not significant.

Direct binding of SNX27 with VPS26 is critical for protecting cargoes from lysosomal degradation^6^, and we hypothesized that FAM21 might regulate cargo recycling via SNX27 binding. We depleted endogenous SNX27 in hTERT-RPE1 cells stably expressing siRNA-resistant wild-type (WT) SNX27 or various mutants (Fig. 3a,b). The SNX27 PDZ domain is required for binding cargoes and VPS26, whereas the FERM domain is required for binding FAM21 (ref. 6; Fig. 3c). As previously reported^6^, lysosomal mis-sorting of GLUT1 by SNX27 depletion was corrected by expression of WT SNX27, but not by the ΔPDZ mutant (Fig. 3d,e and Supplementary Fig. 3a). Intriguingly, expression of the ΔFERM mutant deficient in FAM21 binding resulted in the same defect as FAM21 depletion—mis-sorting of GLUT1 into the Golgi—suggesting that the phenotype is due to a failure in FAM21 binding (Fig. 3d,e and Supplementary Fig. 3b). Even so, we cannot completely exclude the possibility that other interactions of this domain, such as with Ras and some cargo proteins, play a role.

Consistent with a previous report^6^, depletion of SNX27 caused a reduction in GLUT1 levels, which was recovered by treatment of bafilomycin A1, an inhibitor of vacuolar type H^+^-ATPase, indicating that the decrease of GLUT1 was caused by enhanced lysosomal degradation (Fig. 3f). The GLUT1 reduction upon SNX27 depletion was also recovered by expression of WT SNX27, but only partially recovered by the ΔFERM mutant (Fig. 3f), indicating that GLUT1 is not recycled properly and that its lysosomal degradation is still enhanced in the ΔFERM mutant cells. Taken together, these data demonstrate that FAM21 regulates proper recycling of SNX27–retromer cargoes via its interaction with SNX27.

### FAM21 specifies SNX27 localization at endosomal subdomains

**Figure 4:**
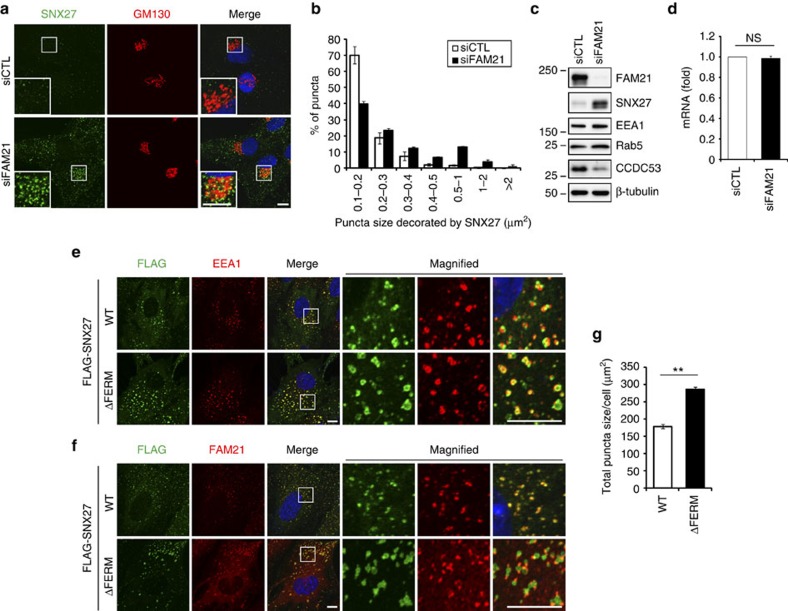
FAM21 regulates a precise localization of SNX27 at an endosomal subdomain through their interaction. (**a**) hTERT-RPE1 cells transfected with the indicated SNX27 or control (siCTL) siRNAs were immunostained for SNX27 (green) and GM130 (red). Insets are magnified views of the Golgi area. Merged images with 4',6-diamidino-2-phenylindole staining (blue) are to the right. (**b**) To measure the size of each punctum, 30 cells of each group were analysed by ImageJ using SNX27 signals from **a**. (**c**) hTERT-RPE1 cells transfected with the indicated siRNAs were immunoblotted. (**d**) The levels of SNX27 mRNA in hTERT-RPE1 cells transfected with the indicated siRNAs were determined by quantitative real-time PCR. (**e**,**f**) hTERT-RPE1 cells stably expressing FLAG-SNX27, WT or ΔFERM mutant were immunostained for FLAG epitope (green) along with EEA1 (**e**, red) or FAM21 (**f**, red). Merged images with 4',6-diamidino-2-phenylindole (blue) staining and magnified views of insets are to the right. (**g**) To measure the total size of puncta visualized by FLAG signals from **e**, 30 cells of each group were analysed with ImageJ. Graphs express means±s.d. **P<0.01; NS, not significant. Scale bars, 10 μm.

We investigated how FAM21 regulates recycling of SNX27–retromer cargoes. First, we examined whether FAM21 depletion affects the localization of SNX27, which serves as a cargo adaptor in the complex. Compared with control cells, the area staining for SNX27 was significantly increased and more concentrated near the Golgi in FAM21-depleted cells (Fig. 4a,b and Supplementary Fig. 4c,d). However, depletion of FAM21 did not alter the puncta size of EEA1, which is associated with the cytoplasmic face of the endosome membrane^30^, indicating that the increase of SNX27 signals is not due to an enlargement of the endosome itself (Supplementary Fig. 4a–c). EEA1 signals were also concentrated near the Golgi in FAM21-depleted cells (Supplementary Fig. 4d), indicating that FAM21 loss leads endosomes to be concentrated in the vicinity of the Golgi. FAM21 depletion did not affect total protein levels of the endosomal marker proteins EEA1 and Rab5, but remarkably increased SNX27 levels, as assessed by immunoblotting (Fig. 4c). A recently uncovered role of FAM21 in modulation of nuclear factor-κB gene transcription led us to examine SNX27 transcript levels^31^. The mRNA level of SNX27 was not affected by FAM21 depletion, suggesting that the increase of SNX27 protein levels would occur in a translational or post-translational manner (Fig. 4d). Together, these results indicate that depletion of FAM21 specifically affects SNX27 levels without interfering with general endosome morphology.

As interaction with FAM21 is critical for SNX27 function, we hypothesized that the increase in SNX27 might be caused by failure to interact with FAM21. We took advantage of cells stably expressing WT SNX27 or the ΔFERM mutant that is deficient in FAM21 binding. WT SNX27 and ΔFERM showed distinct localization patterns within endosomes; WT SNX27 was localized to the subdomain of endosomes stained by EEA1, but exactly co-localized with FAM21 (Fig. 4e,f). In contrast, SNX27 ΔFERM was distributed throughout almost the entire area of endosomes, resulting in an increase of total puncta size as compared with WT (Fig. 4e,g). Specifically, the ΔFERM mutant formed larger, ring-shaped and tubulated endosomes (Fig. 4e, magnified view, green). FAM21 was mostly located at the subdomain or tubules extruding from endosomes decorated by SNX27 ΔFERM (Fig. 4f), consistent with a previous study reporting that actin and WASH complex are specifically located along β2AR-positive tubules on endosomes^32^. Using another cell line expressing a low level of ΔFERM, we confirmed that the entire endosomal distribution of ΔFERM was not due to higher expression than WT (Supplementary Fig. 5). Based on these results, we suggest that SNX27 localizes to a specialized endosomal subdomain via FAM21 interaction to sort cargoes to their correct destination, and that a failure of interaction with FAM21 leads to dispersal of SNX27 throughout the entire surface of endosomes.

### FAM21 maintains proper levels of PI(4)P at the Golgi

**Figure 5:**
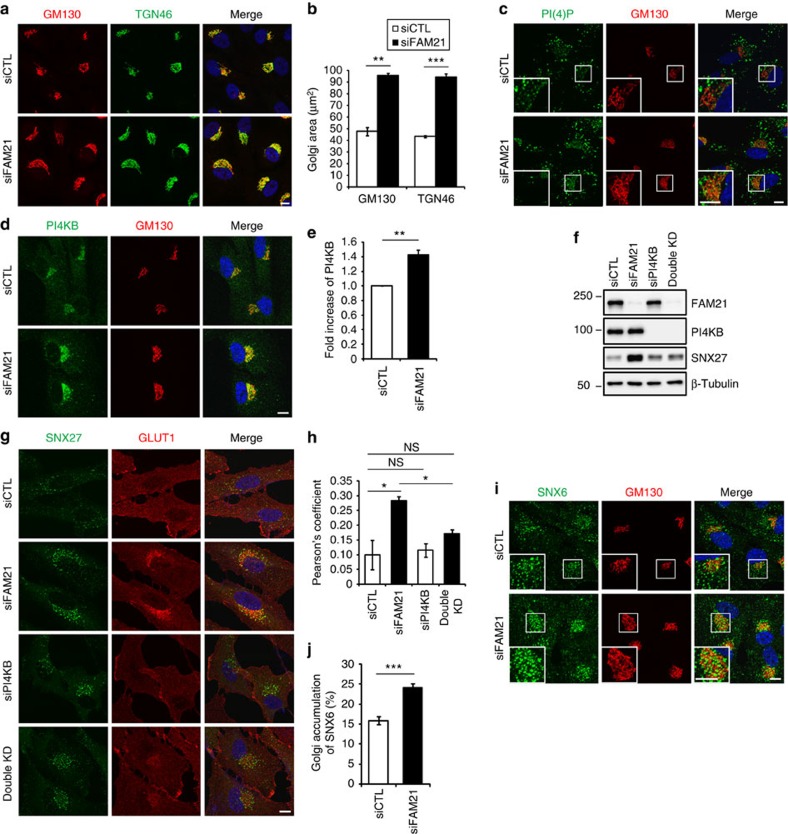
Mis-sorting of GLUT1 into the Golgi in FAM21-depleted cells is caused by an elevation of PI(4)P levels at the Golgi. (**a**) hTERT-RPE1 cells transfected with indicated FAM21 or control (siCTL) siRNAs were immunostained for GM130 (red) and TGN46 (green). (**b**) The area of the Golgi was measured by staining of GM130 or TGN46 from **a**. Graphs express means±s.d. (*n*=3; 20 cells per group). (**c**,**d**) hTERT-RPE1 cells transfected with indicated siRNAs were immunostained for GM130 (red) along with PI(4)P (**c**, green) or PI4KB (**d**, green). (**e**) The intensities of PI4KB at the Golgi were measured from 30 cells of three independent experiments from **d**. Total intensities of PI4KB were divided by the Golgi area. To calculate a fold increase between two groups, averages from FAM21-depleted cells were divided by those of control cells. Graphs express means±s.d. (*n*=3; 30 cells per group). (**f**) hTERT-RPE1 cells transfected with indicated siRNAs were lysed and subjected to immunoblotting. (**g**) hTERT-RPE1 cells transfected with indicated siRNAs were immunostained for SNX27 (green) and GLUT1 (red). (**h**) Co-localization between GLUT1 and GM130 was analysed by calculation of Pearson's coefficient. Graphs express means±s.d. (*n*=3; >30 cells per group). (**i**) hTERT-RPE1 cells transfected with indicated siRNAs were immunostained for SNX6 (green) and GM130 (red). (**j**) The Golgi area was drawn based on GM130 staining, and SNX6 signals in the corresponding area were measured. The area of SNX6 signal per cell was divided by the area of Golgi to calculate the accumulation of SNX6 at the Golgi. Graphs express means±s.d. (20 cells per group). Merged images with 4',6-diamidino-2-phenylindole staining are to the right. Insets are magnified views of the Golgi area. Scale bars, 10 μm. *P<0.05, **P<0.01, ***P<0.001; NS, not significant.

Given that FAM21 depletion causes accumulation of SNX27 and its cargoes at the Golgi, we examined the effect of FAM21 depletion on the Golgi to gain insight into mechanisms of FAM21 in the recycling pathway. When cells were stained for GM130, a *cis*-Golgi matrix protein, and TGN46, a *trans*-Golgi membrane protein, the immunostained Golgi region was expanded in FAM21-depleted cells, to about twofold of that in control cells (Fig. 5a,b).

Each intracellular organelle is identified by a specific subset of activated GTPases and specific lipids, permitting in the proper transport of proteins^33^. PI(4)P, a Golgi-enriched phosphoinositide, facilitates release of retromer-associated cargoes at the TGN^34^. In addition, interactions of the WASH complex with phospholipid and PI(4)P kinase type IIα have been reported^17^,^35^. Therefore, we investigated the relationship of FAM21 with PI(4)P or its kinases. Depletion of FAM21 significantly increased levels of PI(4)P and PI4KB, one of three Golgi-resident PI(4)P kinases, at the Golgi (Fig. 5c–e). The increase of Golgi-localized PI4KB was not accompanied by changes in total PI4KB protein levels, whereas SNX27 protein levels were increased (Fig. 5f).

To examine whether the accumulation of SNX27–retromer cargoes seen in FAM21-depleted cells was caused by an elevation of PI(4)P levels at the Golgi, we depleted PI(4)P in FAM21-depleted cells (Fig. 5f). Transfection of an siRNA targeting PI4KB effectively depleted PI(4)P from the Golgi (Supplementary Fig. 6a). Intriguingly, double knockdown of FAM21 and PI4KB did not induce mis-sorting of GLUT1 (Fig. 5g,h), indicating that the mis-sorting is mediated by increased levels of PI(4)P. Similar to the double knockdown result, treatment of FAM21-depleted cells with PAO, a PI(4)-kinase inhibitor, or PIK93, an inhibitor of PI4KB (ref. 36), abolished GLUT1 accumulation at the Golgi (Supplementary Fig. 6b). These data indicate that GLUT1 mis-sorting defects by FAM21 depletion are caused by an elevation of Golgi PI(4)P levels.

Depletion of PI(4)P causes an accumulation of retromer-associated vesicles in the cytoplasm^34^. We also observed increased levels of SNX27 in PI4KB-depleted cells by immunostaining (Fig. 5g). However, this increase in PI4KB-depleted cells was not accompanied by increased total protein levels of SNX27, distinct from what we observed in FAM21-depleted cells (Fig. 5f). The increase of SNX27 protein level was suppressed by double knockdown (Fig. 5f), suggesting that the increase in SNX27 is also caused by increased levels of PI(4)P.

PI(4)P facilitates dissociation of retromer and its cargoes from the motor at the Golgi by inhibiting the interaction between the p150^Glued^ of the dynein–dynactin complex and SNX6, a cargo adaptor of retromer^34^. Indeed, we observed an enrichment of SNX6 at the Golgi in FAM21-depleted cells, suggesting that the release of the SNX27–retromer cargoes at the Golgi is mediated by dissociation of SNX6 from the motor via increased PI(4)P levels (Fig. 5i,j).

### Distinct function of FAM21 from other WASH components

**Figure 6:**
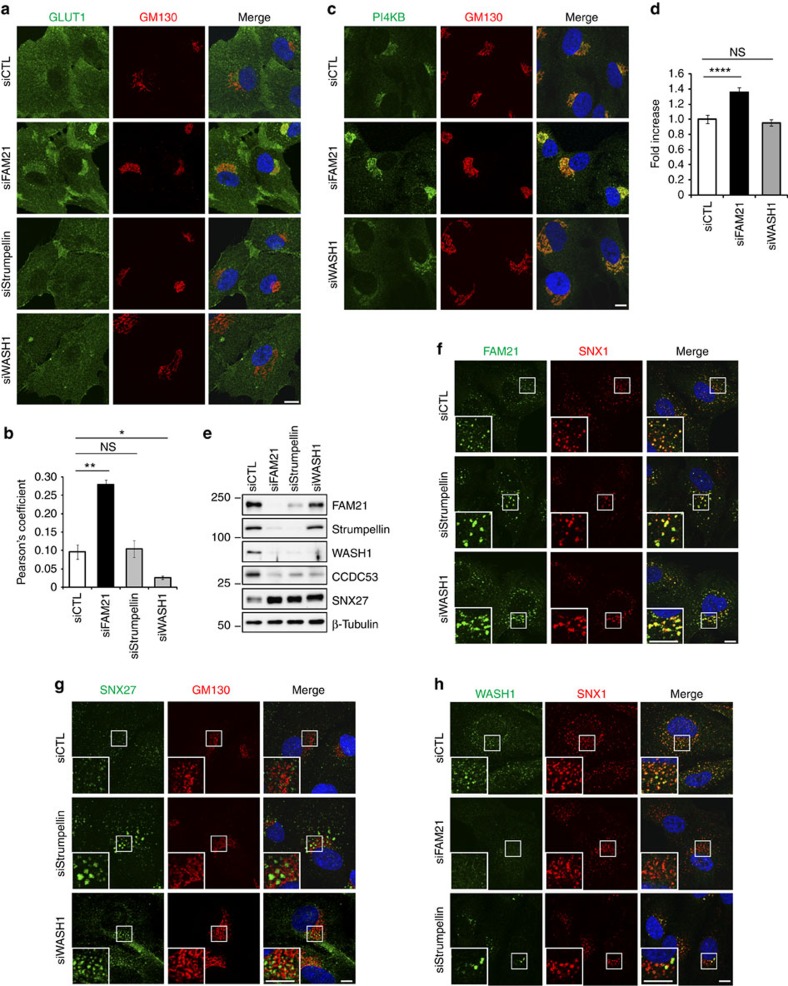
FAM21 has a distinct function in recycling of SNX27–retromer cargoes from other WASH complex components. (**a**) hTERT-RPE1 cells transfected with the indicated specific or control (siCTL) siRNAs were immunostained for GLUT1 (green) and GM130 (red). (**b**) Co-localization between GLUT1 and GM130 was analysed by calculation of Pearson's coefficient. Graphs express means±s.d. (*n*=3; >30 cells per group). (**c**) hTERT-RPE1 cells transfected with indicated siRNAs were immunostained for PI4KB (green) and GM130 (red). (**d**) Intensities of PI4KB at the Golgi were measured from 30 cells per group from **c**. Total intensities of PI4KB were divided by the Golgi area. To calculate a fold-increase from control cells, averages of experimental groups were divided by those of control cells. Graphs express means±s.d. (**e**) hTERT-RPE1 cells transfected with the indicated siRNAs were immunoblotted. (**f**–**h**) hTERT-RPE1 cells transfected with the indicated siRNAs were immunostained as shown. Insets are magnified views of the Golgi area in **g**. Merged images with 4',6-diamidino-2-phenylindole staining are to the right. Scale bars, 10 μm. *P<0.05, **P<0.01, ****P<0.0001; NS, not significant.

As FAM21 is a core component of the WASH complex, we examined whether other WASH complex proteins also function in recycling of SNX27–retromer cargoes. However, cells depleted of the WASH components strumpellin or WASH1 did not accumulate GLUT1 and PI4KB at the Golgi (Fig. 6a–d). The possibility that WASH complex members can function at different steps in the exocytosis pathway was recently raised in a study using *Dictyostelium*^37^. In particular, this study demonstrated that all WASH complex members except FAM21 function to drive actin assembly on lysosomes, whereas FAM21 functions to drive WASH recycling back to lysosomes. Thus, we sought to investigate the distinct phenotypes induced by depletion of FAM21, strumpellin or WASH1 in detail.

Whereas WASH1 and CCDC53 levels were decreased by all three knockdowns, as already demonstrated^17^, levels of FAM21 and strumpellin were not significantly altered by WASH1 depletion (Fig. 6e), and a small amount of FAM21 remained in strumpellin-depleted cells (Fig. 6e). After strumpellin or WASH1 depletion, residual FAM21 was still visible at endosomes, although the FAM21-positive puncta were larger and fewer than in control cells (Fig. 6f). These results demonstrate that FAM21 still localizes to endosomes even in the absence of strumpellin or WASH1, suggesting that the residual FAM21 might be functional in cargo recycling in these cells.

Next, we examined whether SNX27 is affected by depletion of other WASH complex proteins. As in the case of FAM21 depletion, endosomal SNX27 levels were also increased upon depletion of strumpellin or WASH1, with an increase in total protein levels (Fig. 6e,g). Thus, an increase in SNX27 does not always result in GLUT1 and PI4KB accumulation at the Golgi. There are two possible explanations for the above phenotypic differences. The first is that only FAM21 functions in recycling. If so, the increase of SNX27 protein in WASH1- or strumpellin-depleted cells might be caused by a different mechanism than in FAM21-depleted cells. We found that depletion of strumpellin or WASH1 induced enlargement of endosomes, which were enriched with the other WASH complex proteins and SNX1, a component of the retromer complex (Fig. 6f,h and Supplementary Fig. 7). The enlarged and collapsed endosomes seen in these cells were previously observed in WASH-knockout fibroblasts^38^. In contrast, FAM21 depletion did not increase endosome size, but it caused loss of WASH complex proteins from the endosome (Fig. 6h and Supplementary Figs 4a–c and 7). Thus, we cannot exclude the possibility that the increase of SNX27 in strumpellin- or WASH1-depleted cells was caused by general expansion of the endosomal network. A second possible explanation is that FAM21 and the rest of the WASH complex function together in recycling of SNX27–retromer cargoes. In this scenario, FAM21 might regulate PI4KB independently of the WASH complex, but function in SNX27 regulation in collaboration with the WASH complex. As the WASH complex comprises a stable assembly, we favour the latter model. Altogether, we suggest that FAM21 acts as a regulator of membrane protein recycling in conjunction with the SNX27–retromer complex.

## Discussion

**Figure 7:**
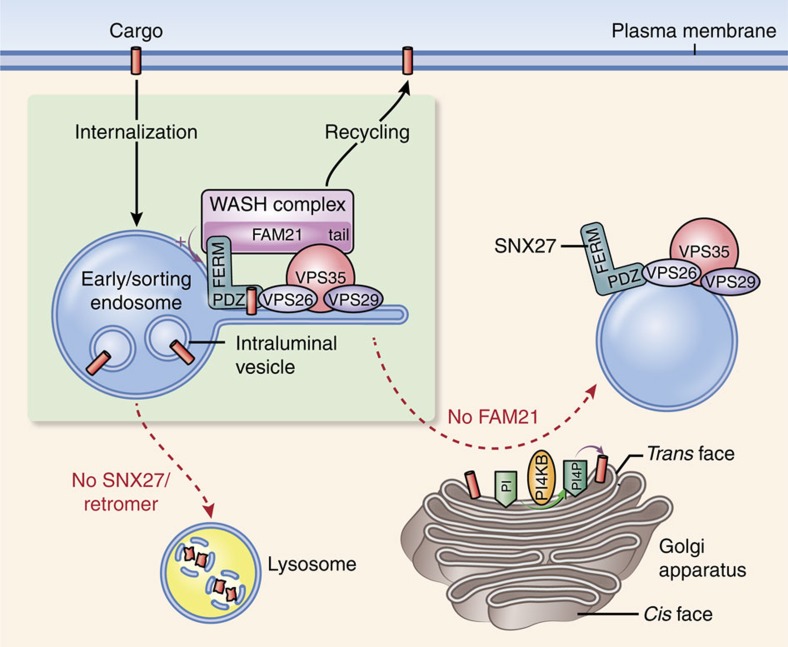
Model for roles of the SNX27–retromer–WASH complex in cargo sorting. After internalization, cargoes harbouring a PDZ-binding motif such as β2AR and GLUT1 bind to a PDZ domain of SNX27. SNX27 serves as an adaptor linking its cargoes to the endosomal tubules through its interaction. Interaction of its PDZ domain with VPS26 of retromer prevents lysosomal entry of cargoes, whereas the interaction of its FERM domain with FAM21 of the WASH complex prevents its transport to the Golgi apparatus. Therefore, these interactions within the SNX27–retromer–WASH complex direct the cargoes towards recycling to the plasma membrane. The WASH complex, in particular, functions in localizing SNX27 at a specialized endosomal subdomain. In addition, FAM21 controls PI4KB levels at the Golgi, resulting in maintaining proper levels of PI(4)P at the Golgi, which prevents dissociation of cargoes from the complex at the Golgi.

Once internalized, membrane proteins are transported to one of three different intracellular compartments from endosomes to meet the needs of cells (Fig. 7). For SNX27–retromer cargoes, trafficking to lysosomes is blocked by the interaction of SNX27 with retromer^6^. Here we show that trafficking of cargoes to the Golgi, another route, is blocked by the interaction of SNX27 with FAM21. The interaction limits SNX27 to a specific endosomal subdomain, which is required for cargo export to the plasma membrane, its destination. Furthermore, FAM21 controls PI(4)P levels at the Golgi to prevent unscheduled dissociation of cargoes from the complex. Therefore, we suggest that the SNX27–retromer–WASH complex acts as a hub to direct cargoes to the plasma membrane by blocking their transport to lysosomes and the Golgi.

Our results indicate that FAM21 might have a distinct role from other WASH components in recycling SNX27–retromer cargoes. FAM21, strumpellin and SWIP are known to regulate the stability of the WASH complex^17^,^22^. Here, we have shown that depletion of strumpellin or WASH1 does not abolish other WASH complex proteins from endosomes, whereas depletion of FAM21 abolishes other proteins (Fig. 6f,h and Supplementary Fig. 7). Our data indicate that FAM21 is the most critical determinant of WASH complex stability. Thus, we infer that residual FAM21 remains functional in strumpellin- or WASH1-depleted cells, so that the accumulation of GLUT1 or PI4KB does not occur. On the other hand, the intact WASH complex appears required for localizing SNX27 to a specific endosomal subdomain, as depletion of any WASH complex protein results in an increase of SNX27 at endosomes (Figs 4 and 6g). Even so, we cannot rule out the possibility that the increase of SNX27 seen in strumpellin- or WASH1-depleted cells is caused by expansion of endosomes^38^ (Fig. 6f,h). In sum, we suggest that the WASH complex functions in limiting SNX27 at the endosomal subdomain, and FAM21 regulates levels of PI4KB at the Golgi independent of the WASH complex. Our suggestion is supported by a recent study showing that FAM21 functions at a different step of exocytosis than other WASH complex components^37^. Also, there are different knockdown phenotypes among FAM21 and other WASH components in endosome size, Golgi morphology and nuclear factor-κB transcriptional activity^22^,^31^,^38^ (Figs 5 and 6 and Supplementary Fig. 4). Thus, growing evidence suggests that FAM21 functions beyond the WASH complex.

Unexpectedly, we found that depletion of FAM21 increases total protein levels of SNX27 (Fig. 4c). As its respective mRNA levels were not changed in FAM21-depleted cells, the increase would not occur at a transcriptional level (Fig. 4d). K63-linked ubiquitination of WASH1 by MAGE-L2-TRIM27, an E3 RING ubiquitin ligase, is known to regulate endosome-to-Golgi and endosome-to-plasma membrane recycling^39^. This finding suggests that proteins which are involved in the endosome-to-plasma membrane recycling pathway could be regulated by ubiquitination. The increase in SNX27 levels was accompanied by increased immunofluorescence intensities at the Golgi (Fig. 4). In the absence of PI4KB, both the increase in SNX27 protein levels and the accumulation of GLUT1, an SNX27 cargo, were suppressed in FAM21-depleted cells (Fig. 5f–h). The results suggest that the increase of protein levels is related to the GLUT1 mis-sorting defect mediated by PI(4)P. As FAM21 is required for limiting SNX27 at the specialized subdomain of endosomes to export its cargoes (Fig. 4), SNX27 would get trapped in endosomes and have prolonged lifetimes in FAM21-depleted cells. We infer that it might result in the increase in SNX27 levels observed in FAM21-depleted cells. However, the mechanism underlying how FAM21 regulates SNX27 and PI4KB should be addressed further.

Mutations in several genes implicated in endosomal sorting pathways are linked to inherited neurological disorders^40^,^41^. VPS35, a subunit of retromer, is mutated in autosomal dominant, late-onset Parkinson disease^42^,^43^. Mutations in WASH components strumpellin or SWIP cause severe, pure, autosomal dominant hereditary spastic paraplegia or autosomal recessive intellectual disability, respectively^44^,^45^,^46^,^47^. It is believed that neurons are more vulnerable to endosomal trafficking defects as neuronal endosomes need to transport signalling molecules through far longer distances than other cell types^48^. Our finding that the SNX27–retromer–WASH complex mainly conducts the endosomal sorting pathway will likely clarify cellular mechanisms underlying these neurological disorders.

## Methods

### DNA constructs

Human SNX27 (GenBank NM_030918), FAM21 (GenBank BC082258), SWIP (GenBank NM_001293640), strumpellin (GenBank NM_014846), WASH1 (GenBank NM_182905) and CCDC53 (GenBank NM_016053) cDNAs were obtained from Open Biosystems. WT and truncated mutants of FAM21 were subcloned into pGW1-Myc vector^49^. WT and truncated mutants of SNX27 were subcloned into pGW1-HA vector or pCDH-CMV-MCS-EF1-Puro-3 × FLAG lentiviral vector (SBI; the sequence for the 3 × FLAG epitope was inserted into *Xba*I/*Eco*RI sites), respectively. siRNA-resistant SNX27 constructs were generated by introducing three silent, mismatched nucleotides (G537A;U540C;G543C) against the siSNX27 target sequence. All constructs were confirmed by DNA sequencing. For detailed information, see Supplementary Table 1.

### Antibodies

The following antibodies were used in the study: anti-FLAG (M2, mouse, Sigma-Aldrich, 1:1,000), anti-GST (B-14, mouse, Santa Cruz Biotechnology, 1:2,000), anti-haemagglutinin (F-7, mouse, Santa Cruz Biotechnology, 1:1,000), anti-*c*-Myc epitope (9E10, mouse, Santa Cruz Biotechnology, 1:1,000), anti-*c*-Myc epitope (polyclonal, goat, Bethyl Laboratories, 1:1,000), anti-His (H-3, mouse, Santa Cruz Biotechnology, 1:500), anti-CBP (W-15, Goat, Santa Cruz Biotechnology, 1:500), anti-GLUT1 (polyclonal, rabbit, Abcam, 1:500), anti-LAMP1 (H4A3, mouse, Abcam, 1:200), anti-GM130 (35, mouse, BD Biosciences, 1:400), anti-GM130 (polyclonal, rabbit, Cell Signaling, 1:400), anti-Rab11 (47, mouse, BD Biosciences, 1:100), anti-SNX27 (1C6, mouse, Abcam, 1:100), anti-β-tubulin (D66, mouse, Sigma-Aldrich, 1:2,000), anti-glyceraldehyde-3-phosphate dehydrogenase (GAPDH; 6C5, mouse, Santa Cruz Biotechnology, 1:2,000), anti-FAM21 (polyclonal, rabbit, EMD Millipore, 1:500), anti-EEA1 (14, mouse, BD Biosciences, 1:200), anti-EEA1 (polyclonal, rabbit, Cell Signaling, 1:200), anti-Rab5 (C8B1, rabbit, Cell Signaling, 1:200), anti-CCDC53 (polyclonal, rabbit, EMD Millipore, 1:200), anti-TGN46 (polyclonal, sheep, AbD Serotec, 1:200), anti-PI(4)P (mouse, Echelon, 1:200), anti-PI4KB (7, mouse, BD Biosciences, 1:200), anti-SNX6 (N-19, goat, Santa Cruz Biotechnology, 1:400), anti-strumpellin (C-14, rabbit, Santa Cruz Biotechnology, 1:200), anti-WASH1 (polyclonal, rabbit, EMD Millipore, 1:400), anti-SNX1 (51, mouse, BD Biosciences, 1:500), anti-Na, K-ATPase (464.6, mouse, Abcam, 1:500) and anti-SWIP (polyclonal, rabbit, EMD Millipore, 1:200).

### Protein purification and *in vitro* pull-down assays

SNX27 cDNA was subcloned into pGEX-6P-1 vector (Amersham) to be fused with GST, and FAM21 cDNA was subcloned into pRSET-A vector (Invitrogen) to be fused with polyhistidine (6 × His). Plasmids were transformed into *E. coli* strain BL21 (DE3) pLysS (Agilent). *E. coli* were grown and induced with 0.2 mM isopropyl-β-D-thiogalactopyranoside at 30 °C for 3 h. Bacterial cells were collected and proteins were purified with Glutathione Sepharose 4B beads (GST fusions; GE Healthcare) or Ni-NTA spin kit (polyhistidine fusions; Qiagen), as per the manufacturer's instructions. After binding, beads were washed three times with binding buffer, and boiled with SDS–PAGE sample buffer. For peptide pull-down assays, peptides corresponding to residues 40–79 (DAGLLQFLQEFSQQTISRTHEIKKQVDGLIRETKATDCRL) and 592–600 (TLCLQAQRE) of FAM21 were synthesized and biotinylated at the N-terminus (GenScript). 1 μg of peptides and 1 μg of bacterially purified CBP-tagged SNX27 proteins were incubated in binding buffer (50 mM Tris-HCl (pH 7.5), 150 mM NaCl, 0.05% Triton X-100) at 4 °C for 16 h, followed by incubation with Streptavidin Sepharose High Performance (GE Healthcare) at 4 °C for an additional 1 h. Beads were washed three times with binding buffer, and bound proteins were analysed by immunoblotting.

### Cell culture, transfection and RNA interference

HEK293T and hTERT-RPE1 cells (American Type Culture Collection) were cultured in DMEM and DMEM/F-12 (Gibco) supplemented with 10% FBS (Gibco) at 37 °C in a 5% CO_2_ humidified incubator, respectively. Cell lines used in the experiments were regularly tested for mycoplasma contamination and treated when appropriate. Cells were transfected with GenJet Plus (SignaGen Laboratories) or Avalanche-Omni (EZ Biosystems) for plasmid DNAs, and Lipofectamine RNAiMax (Invitrogen) for siRNAs as per the manufacturer's instructions. The following sequences of siRNAs were used: siFAM21-1 (Ambion): 5′- GAACAAAACCCAAGGCAAA -3′; siFAM21-2 (Ambion): 5′- GAGUGAAGUCUGUGGAUAA -3′; siFAM21-3 (Ambion): 5′- GGACAGUGCCUUUGAGCAA -3′; siSNX27 (Invitrogen): 5′- GGUGAGAAGUUUGUGGUAUAUAAUG -3′; siPI4KB (Invitrogen): 5′- GCUCCUGAGAGAGAAUUCAUCAAGU -3′; siStrumpellin (Dharmacon): 5′- GGAUGAGUCUGUAACGUUU -3′; siWASH1 (Ambion): 5′- ACUACUUCUAUGUGCCAGA -3′. Control siRNAs were obtained from Ambion.

### Generation of stable cell lines

hTERT-RPE1 cells stably expressing SNX27 WT or mutants were generated by lentiviral infection^50^. pCMV-dR8.2 dvpr and pCMV-VSV-G (Addgene) were co-transfected into HEK293T cells along with respective pCDH-CMV-MCS-EF1-Puro-based constructs. The next day, lentivirus was harvested from the culture media by centrifugation and frozen at −80 °C. hTERT-RPE1 cells were infected with the indicated viruses, and 2 days later infected cells were incubated with 20 μg ml^−1^ of puromycin for selection for an additional 2 days.

### Chemicals

Dimethylsulphoxide (D8418), BFA (B5936), PAO (P3075) and PIK93 (SML0546) were obtained from Sigma-Aldrich. Bafilomycin A1 (19-148) was purchased from EMD Millipore.

### Immunofluorescence staining and confocal microscopy

hTERT-RPE1 cells seeded on coverslips were fixed with 4% paraformaldehyde for 15 min. Fixed cells were blocked with 3% normal donkey serum in PBST (PBS with 0.1% Triton X-100) or PBSS (PBS with 0.05% saponin) for 15 min. Then, cells were incubated with primary antibodies diluted in blocking solution for 2 h, washed four times with PBST or PBSS, incubated with Alexa Fluor-conjugated secondary antibodies (Molecular Probes) diluted in blocking solution for 30 min, and washed with PBST or PBSS four times. For the Tf uptake assay, cells were incubated with 10 μg ml^−1^ of Alexa 568-conjugated Tf (Molecular Probes) for 20 min at 37 °C before fixation. PI(4)P staining was performed as described previously^51^. Cells were fixed with 2% paraformaldehyde for 15 min, washed with PBS containing 50 mM NH_4_Cl and permeabilized with 20 μM digitonin in Buffer A (20 mM pipes (pH 6.8), 137 mM NaCl and 2.7 mM KCl) for 5 min. After washing with Buffer A, cells were blocked with blocking solution (5% normal donkey serum in Buffer A) with 50 mM NH_4_Cl for 45 min. Then, cells were incubated with primary antibodies diluted in blocking solution for 2 h, washed with Buffer A and incubated with secondary antibodies diluted in blocking solution for 45 min. For nuclear staining, 4′,6-diamidino-2-phenylindole (Molecular Probes) was applied for 5 min. Cells were mounted on a slide glass using Fluoromount-G (SouthernBiotech). Cells were visualized using a Zeiss LSM710 confocal microscope with a × 63, 1.4 numerical aperture Plan-Apochromat lens. Images were acquired and processed using ZEN 2009 (Carl Zeiss) and ImageJ (NIH) software.

### Quantification of co-localization and statistical analysis

Co-localization analysis was performed on the confocal sections showing maximum signals of GLUT1, PI4KB or Na,K-ATPase using ImageJ (NIH). JACoP (Just another co-localization plugin) was used to calculate the Pearson's coefficient from more than 30 cells per group, from three independent experiments^52^. Microsoft Excel 2007 was used to analyse statistical data and to draw graphs. Significance levels for comparisons between groups were determined by Student's *t*-tests, and *P*<0.05 was considered significant. The variance was similar between the groups we statistically compared.

### Immunoprecipitation and immunoblotting

Immunoprecipitation and immunoblotting were performed as described previously^53^. In detail, cells were washed with PBS and then lysed in lysis buffer (50 mM Tris-HCl (pH 8.0), 150 mM NaCl, 1% Triton X-100, 5 mM EGTA (pH 8.0), 1.5 mM EDTA (pH 8.0), Complete Protease Inhibitor Cocktail (Roche)) on ice for 30 min. Lysates were clarified by centrifugation (14,000*g*) for 20 min at 4 °C, and incubated with antibodies at 4 °C overnight. Protein A/G PLUS-agarose beads were added for 1 h, and then the beads were washed three times with lysis buffer. Bound proteins were resolved by SDS–PAGE and then electrophoretically transferred to nitrocellulose membranes. Membranes were blocked with 5% nonfat milk in Tris-buffered saline with 0.05% Tween-20 (TBST) for 30 min, and incubated with primary antibodies in 3% BSA in TBST at 4 °C overnight. Blots were washed three times with TBST, incubated with horseradish peroxidase-conjugated secondary antibodies (Santa Cruz Biotechnology, 1:2,000) for 1 h, and then washed three times with TBST. Immun-Star WesternC (Bio-Rad) or Amersham ECL Prime (GE Healthcare) was used to detect immunoreactive proteins. Images were obtained and processed using a ChemiDoc XRS+ System with Image Lab software (Bio-Rad). Uncropped blot images are presented in Supplementary Fig. 8.

### Quantitative real-time PCR (qRT–PCR)

RNA was extracted using the RNeasy Mini Kit (Qiagen) and cDNAs were obtained using SuperScript III First-Strand Synthesis SuperMix for qRT–PCR system (Invitrogen). qRT–PCR was performed with SYBR Green PCR Master Mix (Invitrogen) using Applied Biosystems StepOnePlus System (Applied Biosystems). Experiments were performed in quadruplicate using three independent cDNAs. The qRT–PCR results were normalized to GAPDH and calculated following the 2^−ΔΔCt^ method^54^. The following sequences of primers were used: GAPDH-F: 5′- GGTCGGAGTCAACGGATTTGGTCG -3′; GAPDH-R: 5′- CCTCCGACGCCTGCTTCACCAC -3′; SNX27-F: 5′- GAGCAGGCGAGAAGGAATTG -3′; SNX27-R: 5′- GCTTAGAACACAGCTGCCTC -3′.

## Additional information

**How to cite this article:** Lee, S. *et al*. FAM21 directs SNX27–retromer cargoes to the plasma membrane by preventing transport to the Golgi apparatus. *Nat. Commun.* 7:10939 doi: 10.1038/ncomms10939 (2016).

## Supplementary Material

Supplementary Information Supplementary Figures 1-8 and Supplementary Table 1
