# Supplementary material for: FAM21 directs SNX27–retromer cargoes to the plasma membrane by preventing transport to the Golgi apparatus
Source: Nat Commun. 2016 Mar 9;7:10939. doi: 10.1038/ncomms10939 (PMC4786876; doi:10.1038/ncomms10939)
Supplement: Supplementary Information — Supplementary Figures 1-8 and Supplementary Table 1 [file ncomms10939-s1.pdf]

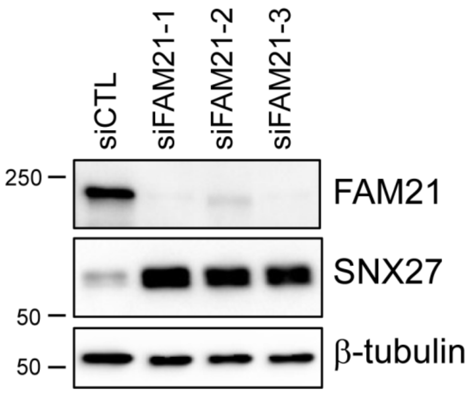

Supplementary Figure 1 | Three different siRNA sequences targeting FAM21 effectively deplete endogenous FAM21. hTERT-RPE1 cells transfected with the indicated siRNAs were lysed and immunoblotted as shown. Migrations of molecular weight markers (in kDa) are at the left.

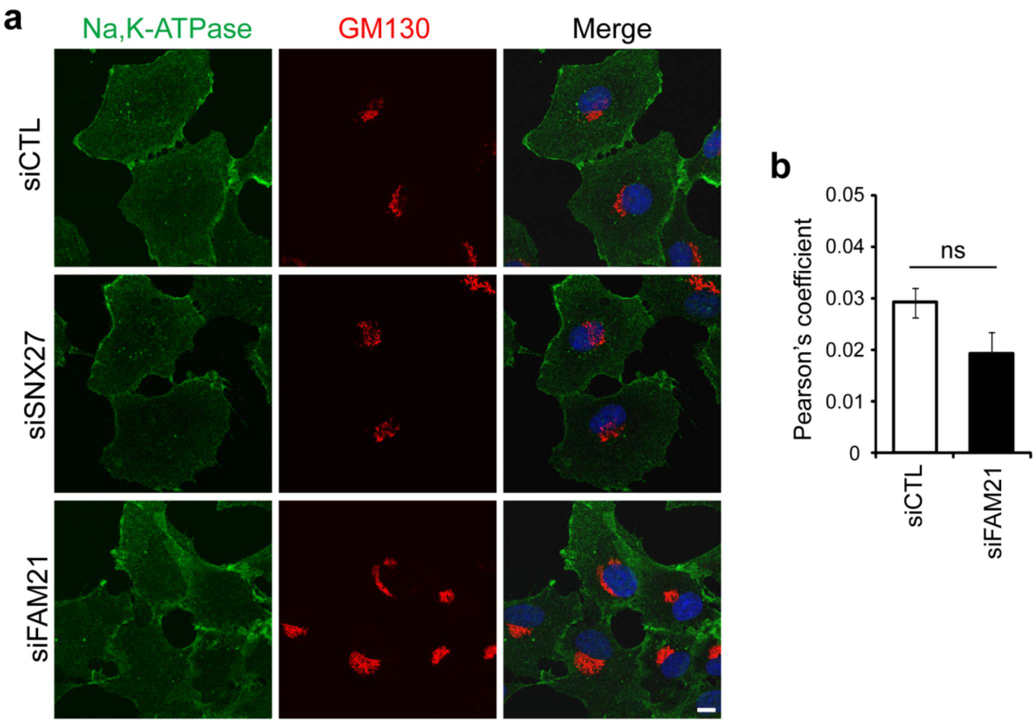

Supplementary Figure 2 | Regulation of recycling by FAM21 is unique to membrane proteins that interact with SNX27. (**a**) hTERT-RPE1 cells transfected with the indicated siRNAs were immunostained for Na,K-ATPase (green) and GM130 (red). Merged images with DAPI staining are to the right. Scale bar, 10 µm. (**b**) Co-localizations of Na,K-ATPase and GM130 were analyzed by calculation of Pearson's coefficient. Graphs express means ± s.d. (30 cells per group). ns, not significant.

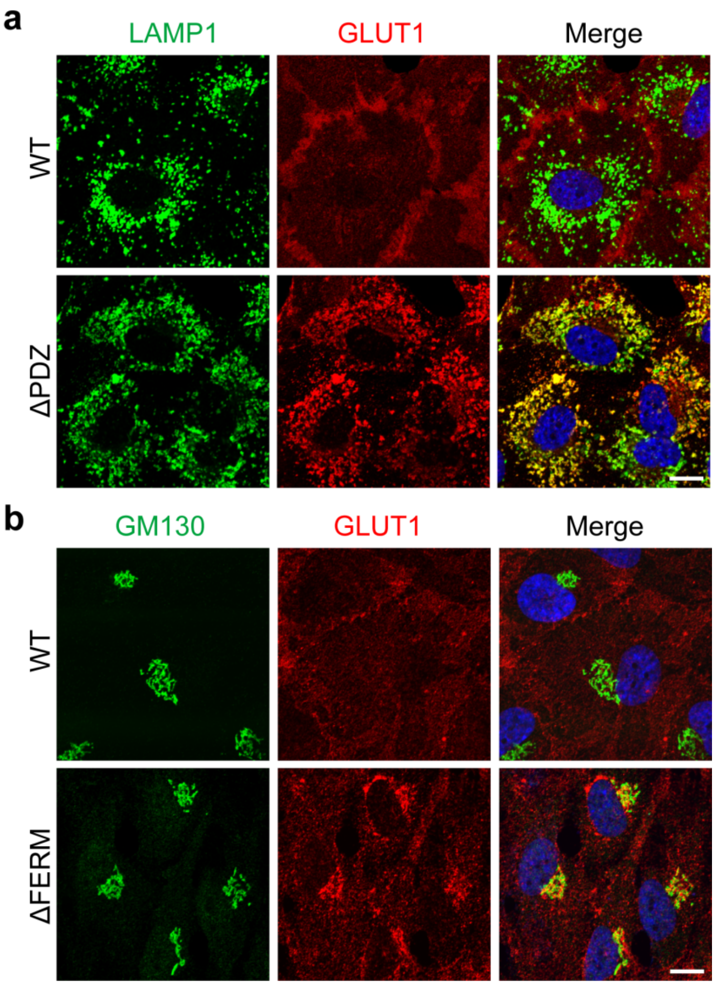

Supplementary Figure 3 | SNX27 ΔPDZ and ΔFERM mutants cannot rescue GLUT1 mis-sorting induced by SNX27 depletion. (**a**,**b**) hTERT-RPE1 cells stably expressing the indicated 3×FLAG-tagged, siRNA-resistant SNX27 constructs were transfected with an siRNA targeting SNX27 and then immunostained for GLUT1 (red) along with LAMP1 (**a**, green) or GM130 (**b**, green). Merged images with DAPI staining (blue) are to the right. Scale bars, 10 μm.

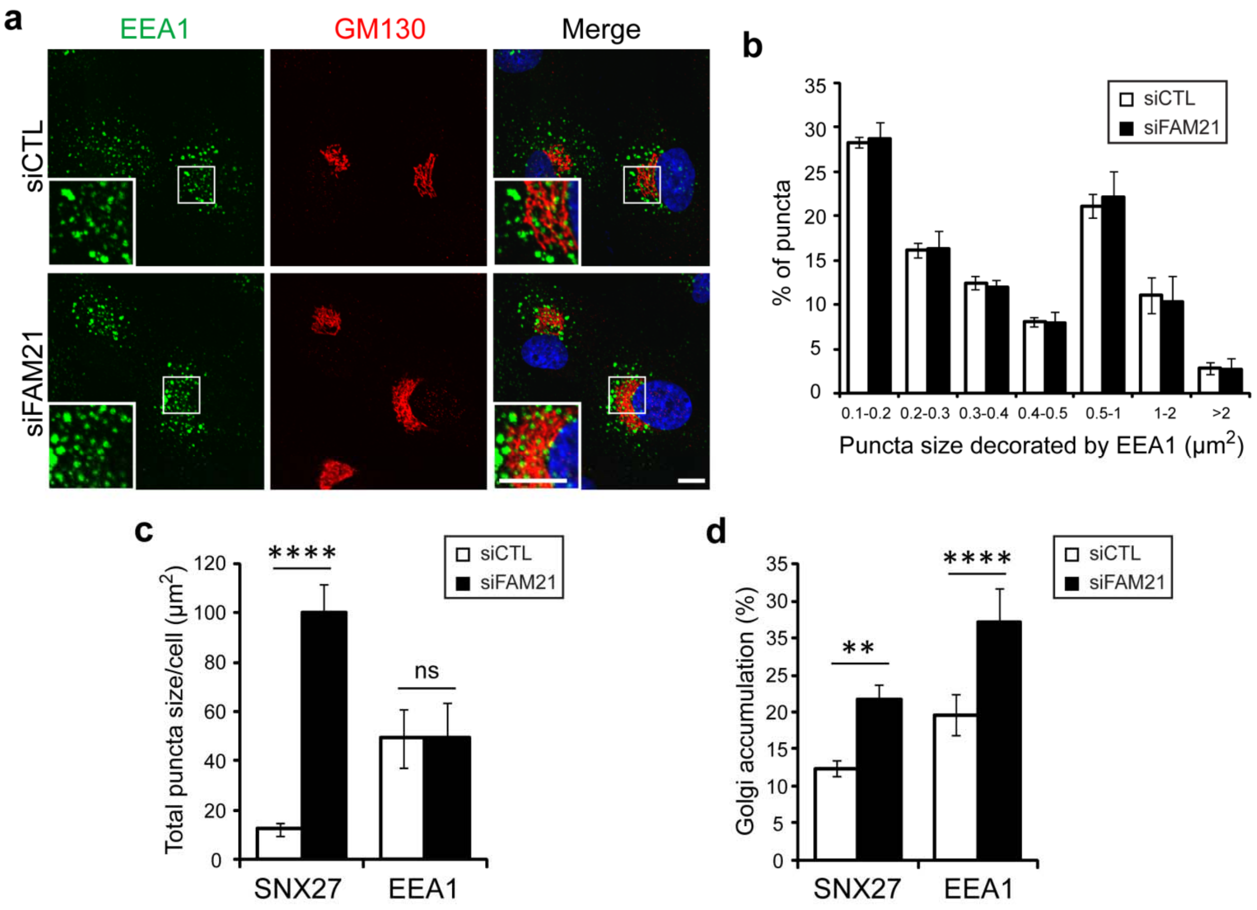

Supplementary Figure 4 | Depletion of FAM21 does not affect endosome size, but affects endosome distribution. (**a**) hTERT-RPE1 cells transfected with the indicated siRNAs were immunostained for EEA1 (green) and GM130 (red). Insets are magnified views of the Golgi area. Merged images with DAPI staining (blue) are to the right. Scale bars, 10 μm. (**b**) To measure the size of each punctum, 30 cells from each group were analyzed with ImageJ based on EEA1 signals from **a**. (**c**) Total puncta area within each cell was calculated for SNX27 (Fig. 4b) and EEA1 (**b**). (**d**) Populations of SNX27 (Fig. 4a) and EEA1 (**a**) localized at the Golgi were calculated by dividing signals of the respective proteins in the Golgi area by total signals within each cell. Graphs express means ± s.d. (30 cells per group). $^{**}P < 0.01$, $^{****}P < 0.0001$; ns, not significant.

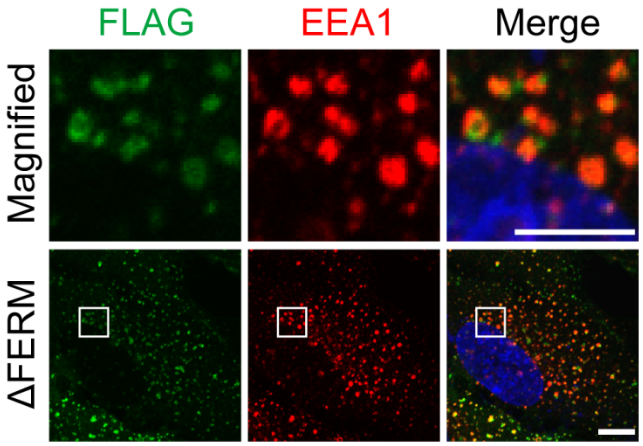

Supplementary Figure 5 | SNX27 ΔFERM mutant is localized across the entire area of endosomes even when expressed at low levels. hTERT-RPE1 cells stably expressing the FLAG-SNX27 ΔFERM mutant were generated with a lower titer of lentivirus than used in Fig. 4. Cells were immunostained for FLAG (green) and EEA1 (red). Magnified views of boxed areas in the lower panels are at the top. Scale bars, 10 μm; 5 μm (magnified).

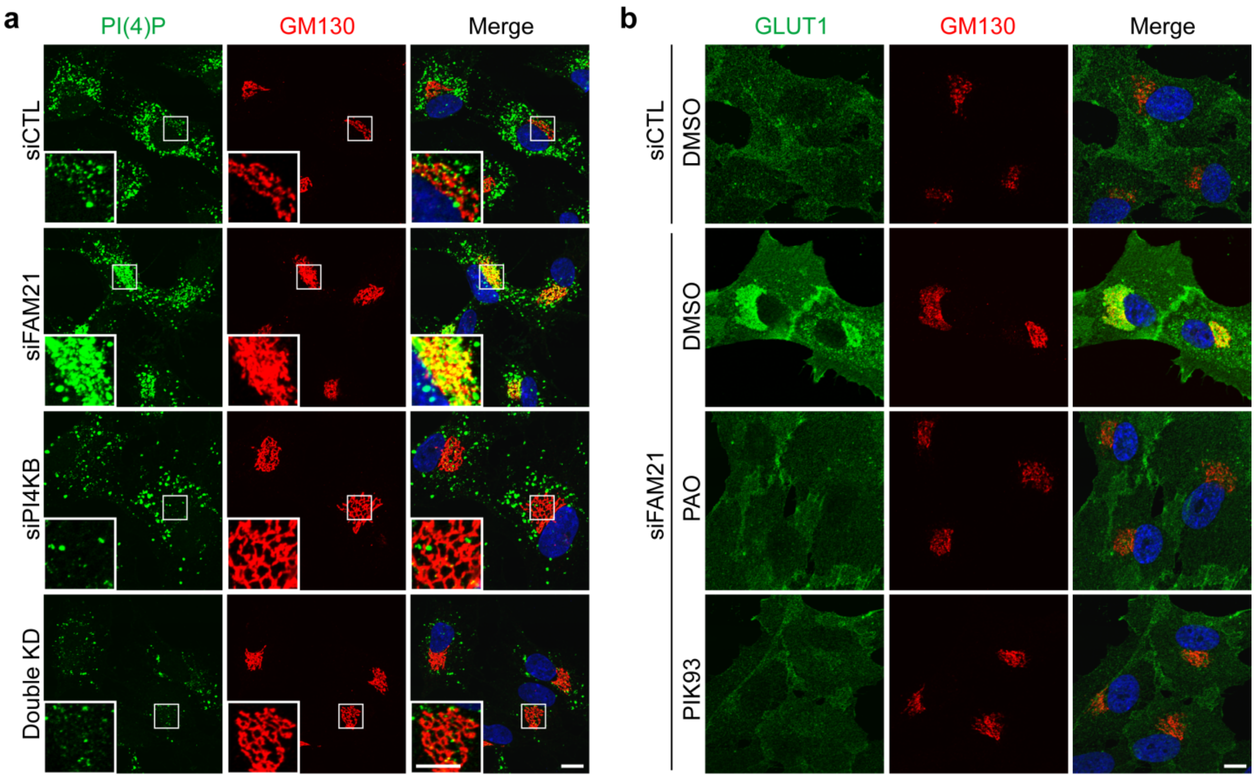

Supplementary Figure 6 | Mis-sorting of GLUT1 into the Golgi apparatus in FAM21-depleted cells is recovered by treatment with PI(4)-kinase inhibitors. (**a**) hTERT-RPE1 cells transfected with the indicated siRNAs were co-immunostained for PI(4)P (green) and GM130 (red). Insets are magnified views of the Golgi area. (**b**) hTERT-RPE1 cells were transfected with the indicated siRNAs. Cells were treated at 37 °C with 0.1% DMSO for 2 h, 20 μM of PAO for 20 min or 0.5 μM of PIK93 for 2 h before fixation. Fixed cells were immunostained for GLUT1 (green) and GM130 (red). Merged images with DAPI staining (blue) are to the right. Scale bars, 10 μm.

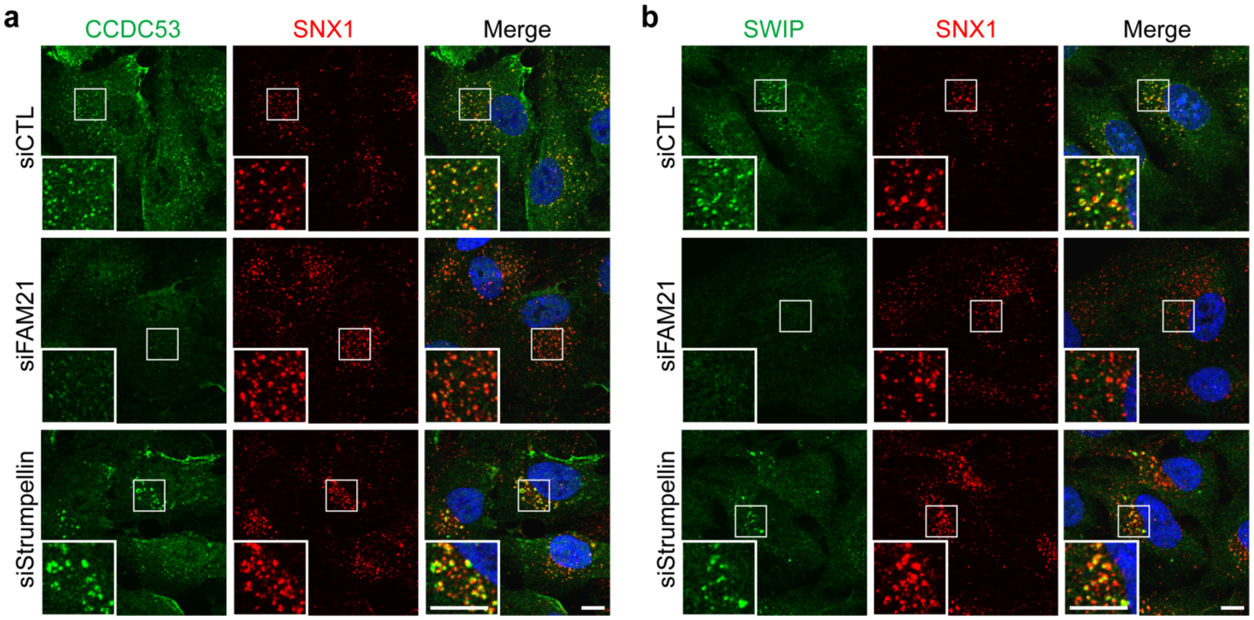

Supplementary Figure 7 | WASH complex proteins are eliminated from endosomes upon FAM21 depletion, but not by strumpellin depletion. (**a**,**b**) hTERT-RPE1 cells transfected with the indicated siRNAs were co-immunostained for SNX1 (red) along with CCDC53 (**a**, green) or SWIP (**b**, green). Merged images with DAPI staining are to the right. Scale bars, 10 μm.

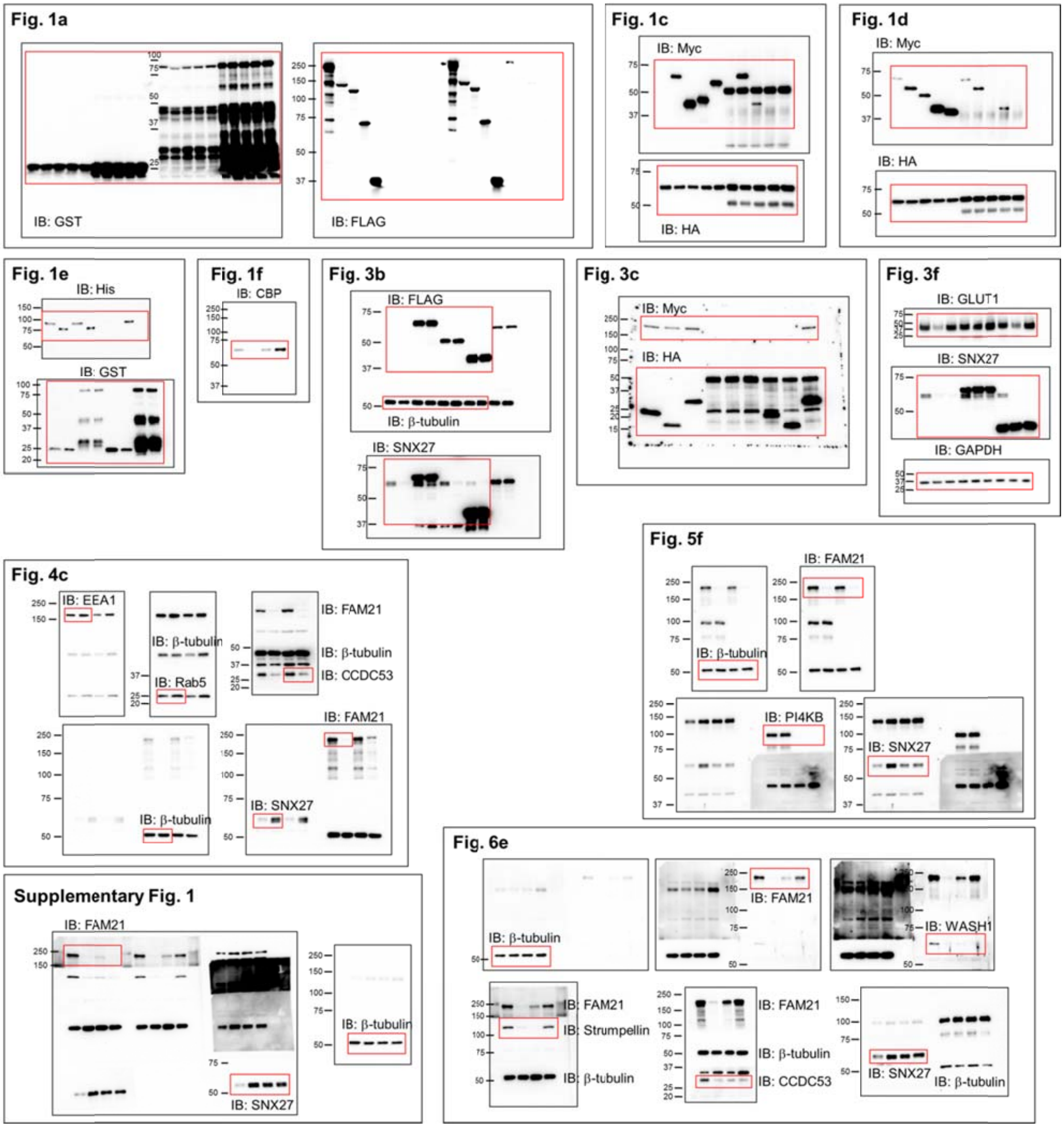

Supplementary Figure 8 | Uncropped images of immunoblots. Red rectangles delineate the regions included in the indicated figures.

Supplementary Table 1. List of DNA constructs used in the study.

Insert	Vector	Cloning site
SNX27	pGEX-6P-1	BamHI/XhoI
SNX27	pGW1-HA	AscI/SalI
SNX27	p-CAL-n-FLAG	BamHI/XhoI
SNX27 (1-155)	pGW1-HA	EcoRI
SNX27 (156-268)	pGW1-HA	XmaI
SNX27 (270-528)	pGW1-HA	XmaI
SNX27-r	pCDH-CMV-MCS-EF1-Puro-3XFLAG	BamHI
SNX27 ∆PDZ (135-528)-r	pCDH-CMV-MCS-EF1-Puro-3XFLAG	BamHI
SNX27 ∆FERM (1-273)-r	pCDH-CMV-MCS-EF1-Puro-3XFLAG	BamHI/NotI
FAM21	pGW1-Myc	AscI/SalI
FAM21 (1-356)	pGW1-Myc	AscI/SalI
FAM21 (356-600)	pGW1-Myc	AscI/SalI
FAM21 (601-900)	pGW1-Myc	AscI/SalI
FAM21 (901-1341)	pGW1-Myc	AscI/EcoRI
FAM21 (40-356)	pGW1-Myc	AscI/EcoRI
FAM21 (80-356)	pGW1-Myc	AscI/EcoRI
FAM21 (356-591)	pGW1-Myc	AscI/EcoRI
FAM21 (1-600)	pRSET-A	BamHI/PstI
FAM21 (80-591)	pRSET-A	BamHI/PstI
FAM21	pGW1-3XFLAG	AscI/SalI
SWIP	pGW1-3XFLAG	AscI/XmaI
Strumpellin	pGW1-3XFLAG	XmaI
WASH1	pGW1-3XFLAG	AscI/XmaI
CCDC53	pGW1-3XFLAG	EcoRI

Numbers indicate amino acids
'r' means siRNA-resistant
